# Bioluminescence-Based High-Throughput Screen Identifies Pharmacological Agents That Target Neurotransmitter Signaling in Small Cell Lung Carcinoma

**DOI:** 10.1371/journal.pone.0024132

**Published:** 2011-09-08

**Authors:** Ma. Reina D. Improgo, Christopher W. Johnson, Andrew R. Tapper, Paul D. Gardner

**Affiliations:** Brudnick Neuropsychiatric Research Institute, Department of Psychiatry, University of Massachusetts Medical School, Worcester, Massachusetts, United States of America; City of Hope National Medical Center and Beckman Research Institute, United States of America

## Abstract

**Background:**

Frontline treatment of small cell lung carcinoma (SCLC) relies heavily on chemotherapeutic agents and radiation therapy. Though SCLC patients respond well to initial cycles of chemotherapy, they eventually develop resistance. Identification of novel therapies against SCLC is therefore imperative.

**Methods and Findings:**

We have designed a bioluminescence-based cell viability assay for high-throughput screening of anti-SCLC agents. The assay was first validated via standard pharmacological agents and RNA interference using two human SCLC cell lines. We then utilized the assay in a high-throughput screen using the LOPAC^1280^ compound library. The screening identified several drugs that target classic cancer signaling pathways as well as neuroendocrine markers in SCLC. In particular, perturbation of dopaminergic and serotonergic signaling inhibits SCLC cell viability.

**Conclusions:**

The convergence of our pharmacological data with key SCLC pathway components reiterates the importance of neurotransmitter signaling in SCLC etiology and points to possible leads for drug development.

## Introduction

Lung cancer is the leading cause of cancer-related mortality worldwide, resulting in over 1.3 million deaths per year [1]. In the United States, lung cancer incidence rates are second only to rates for breast cancer in females and prostate cancer in males [2]. Tobacco use is the major risk factor associated with lung cancer. Histopathological classification divides lung cancer into two main types: small cell lung carcinoma (SCLC) and non-small cell lung carcinoma (NSCLC). NSCLC can be further subdivided into adenocarcinoma, squamous cell, and large cell lung carcinoma.

SCLC displays the most aggressive clinical progression of any type of lung cancer, as demonstrated by its rapid doubling time and early development of widespread metastases [3]. In fact, SCLC is so aggressive that by the time it is diagnosed, metastasis has usually already occurred such that surgical resection of tumors is rarely an option. Hence, chemotherapy and radiation are the treatments of choice for these patients. Most patients exhibit robust initial response to treatment but eventually become chemoresistant [4]. Relapses occur almost without exception and five-year survival rates range from 31% (for patients diagnosed at Stage I) to 2% (for patients diagnosed at Stage IV) [2]. Advances made in the past three decades have resulted in only a slight improvement in treatment outcome for SCLC [5]. Identification of novel SCLC therapies is therefore of prime importance.

Cell viability assays are indispensable tools in drug discovery efforts. Measurement of cell viability is a simple and rapid approach for determining a cell population's response to endogenous factors such as hormones and growth factors as well as external stimuli such as drugs and environmental stress [6]. A classic approach for measuring cell viability involves the use of vital dyes (e.g., trypan blue) for probing membrane integrity. This method, however, is tedious and prone to experimenter bias [6]. Another traditional method relies on the reduction of tetrazolium salts such as MTT (3-(4,5-dimethylthiazolyl-2)-2,5-diphenyltetrazolium bromide), resulting in the formation of colored products that can be quantified via spectrophotometry [7]. However, such assays have limited sensitivity, narrow dynamic ranges, and are subject to variability [6].

Bioluminescence-based assays are a favored approach due to their broad linearity and robustness to library compounds and complex biological samples [8]. These assays exploit the ability of luciferase to catalyze oxidation of the luciferin substrate, a reaction that generates light as a by-product [8]. Light generated by this reaction has the highest quantum efficiency of any known chemiluminescent reaction [9]. Combined with low bioluminescence signals in mammalian cells, this approach allows for highly sensitive assays.

Here, we developed a cell viability assay employing bioluminescence to screen for pharmacological compounds against SCLC. From a library of 1,280 pharmacologically active compounds, we identified several classes of drugs that target classic cancer signaling pathways as well as neuroendocrine markers in SCLC.

## Materials and Methods

### Ethics Statement

All animal experiments were conducted in accordance with the guidelines for care and use of laboratory animals provided by the National Research Council [10], as well as with an approved animal protocol from the Institutional Animal Care and Use Committee of the University of Massachusetts Medical School (Assurance Number A-3306-01). Specifically, mice were exposed to 2% isofluorane before being imaged. During imaging, mice lay on a temperature-regulated stage and were continually exposed to isofluorane.

### Cell culture

DMS-53 and DMS-114 SCLC cell lines were acquired from the American Type Culture Collection (ATCC) and grown in RPMI 1640 containing 2 mM L-glutamine and 25 mM HEPES (Cellgro), supplemented with 10% fetal bovine serum (PAA). HEK293T cells were acquired from Open Biosystems and grown in Dulbecco's Modified Eagle's Medium containing 4 mM L-glutamine and 4.5 g/L glucose (Cellgro), supplemented with 10% fetal bovine serum. Cells were maintained at 37°C and 8% CO_2_. Cell line authentication is performed by the American Type Culture Collection using cytochrome oxidase subunit I (COI) analysis for interspecies identification and STR analysis (DNA profiling) for intraspecies identification.

### Cloning and Virus Production

A luciferase cassette was subcloned from pGL3-Basic (Promega) into the multiple cloning site of the lentiviral expression vector pLEX-MCS (Open Biosystems) using *SpeI* and *MluI* (New England Biolabs) restriction sites. The construct, pLEX-lucSM, was transfected into HEK293T cells for viral packaging using the Trans-Lentiviral Packaging System (Open Biosystems). Viral particles were harvested and used to transduce DMS-53 or DMS-114 cells in the presence of 4 µg/mL polybrene (Sigma). To select for cells stably expressing luciferase (designated DMS-53 luc+ and DMS-114 luc+), cells were treated with 6 µg/mL puromycin dihydrochloride for 5 days.

### Luciferase Assays

Cells were lysed using 50 µL Reporter Lysis Buffer (Promega) and placed on a shaker at room temperature for 5 minutes. To snap-freeze, cells were placed at −80°C for 15 minutes. Cells were then allowed to thaw and equilibrate to room temperature for 15 minutes. Plates were returned to the shaker for another 5 minutes before placing into a luminometer (Bio-Rad Lumimark). The luminometer was set to dispense 50 µL of the luciferase substrate (Promega Luciferase Assay Reagent). Integration time was set for 10 seconds with a 2-second lag time. Non-luciferase expressing cells were used as negative controls, where indicated.

### MTT Assay

Cells were seeded from 0–1×10^6^ cells/well in black, clear bottom 96-well assay plates and allowed to grow overnight. Cells were then treated with 10 µL MTT Reagent (ATCC) and incubated for 4 hours. After ensuring that purple precipitates were visible, 100 µL of Detergent Reagent (ATCC) was added. Samples were allowed to incubate at room temperature for another 2 hours. Absorbance readings at 570 nm were taken using a SpectraMax M2 microplate reader (Molecular Devices).

### Bioluminescence Imaging

For imaging of luciferase-expressing cells *in vitro*, cells were seeded onto black, clear bottom 96-well assay plates (Costar). Before imaging, cell culture media were removed. The firefly luciferase substrate D-luciferin (Gold Biotechnology) was added at a final concentration of 150 µg/mL per well. After 15 minutes of incubation, cells were imaged using a Xenogen IVIS 100 imager (Caliper Life Sciences), which makes use of a supercooled charge-coupled device (CCD) camera to detect light-emitting cells. For *in vivo* work, male athymic nude mice were obtained from Charles River Laboratories. For xenograft assays, cells were implanted subcutaneously into the hind flanks of 6-week old mice. For the lung colonization model, cells were injected into tail veins of 6-week old mice. Mice were injected intraperitoneally with 150 mg/kg D-luciferin 15 minutes prior to imaging. Quantification was performed using the acquisition and analysis software Living Image (Caliper Life Sciences).

### Pharmacological Treatments

All drugs were purchased from Sigma. For pre-validation of the bioluminescence assay, 1×10^4^ DMS-53 luc+ and DMS-114 luc+ cells were seeded in black, clear bottom 96-well assay plates and allowed to grow overnight. Cells were treated with 0, 2, and 4 µM K252c (staurosporine aglycone) for 0, 12, and 24 hours or 0, 25, and 50 µM cis-diammineplatinum (II) chloride (cisplatin) for 0, 12, and 24 hours. Cells were then harvested and subjected to luciferase assays.

### RNA Interference

Cells were seeded in black, clear bottom 96-well assay plates and allowed to grow overnight. Cells were transfected with 5–10 nM of a Silencer Select Negative Control #1 or glyceraldehyde 3-phosphate dehydrogenase (GAPDH) siRNA (Applied Biosystems) using Lipofectamine 2000 (Invitrogen) in Opti-MEM (Invitrogen). Samples treated only with Lipofectamine 2000 were also used as controls. After 48 hours, cells were harvested and subjected to luciferase assays. To determine knockdown efficiency, cells were seeded in parallel onto 6-well cluster plates and transfected as above. After 48 hours, cells were harvested and total RNA was isolated using an RNeasy Mini Kit (Qiagen). RNA was reverse-transcribed using RETROscript reagents (Applied Biosystems). Samples without reverse transcriptase were used as negative controls. GAPDH amplicons were generated using GAPDH TaqMan assays (Applied Biosystems) and the PRISM 7500 real-time PCR system (Applied Biosystems). GAPDH levels were quantified using the 2^−ΔΔCt^ method [11]. ß2-microglobulin was used as the endogenous control to normalize gene expression levels.

### Large-Scale Compound Screen

For primary screening, 5×10^6^ DMS-53+ cells were seeded in black, clear bottom 96-well assay plates and allowed to grow overnight. The following day, compounds from the Library of Pharmacologically Active Compounds, LOPAC^1280^ (Sigma), were added to each well (final concentration = 50 µM in 1% DMSO). For each plate, one column of cells (n = 8 wells) was treated for 24 hours with equal concentrations of cisplatin as positive control and another column was treated with 1% DMSO as negative control. Tolerance of cells for 1% DMSO was confirmed prior to screening (Fig. S1). Media aspiration and addition of compounds, lysis buffer, and luciferase substrate were performed with a Te-Mo (Tecan) automated system at the University of Massachusetts Medical School Small Molecule Screening Facility. Luciferase readouts were taken using a Victor plate reader (Perkin Elmer). For secondary screening, selected hits from the primary screen were retested using DMS-53 luc+ cells and further confirmed using DMS-114 luc+ cells. For tertiary verification, DMS-53 luc+ cells were treated with increasing doses (0, 25, 50, and 100 µM) of the representative drugs cortexolone maleate/ST-148 (Sigma) and fluoxetine hydrochloride (Sigma) for 24 hours, followed by luciferase assays.

### Analysis

Assay quality was measured using three statistical parameters [12]. Signal-to-background ratios (S/B) were calculated using the equation: S/B = μ_max_/μ_min_. Signal-to-noise ratios (S/N) were calculated using the equation: S/N = (μ_max_−μ_min_)/σ_min_ of treated controls. For S/B and S/N, values >2 are considered acceptable. Z′-factor values were calculated using the equation Z′ factor = 1−(3σ_max_+3σ_min_)/|μ_max_−μ_min_|. For all equations, μ represents means and σ represent standard deviations (SD). For Z′-factor interpretation, we used the scale developed by Zhang and colleagues [13], wherein a score of 1.0 is considered ideal; scores between 0.5 and 1.0 represent excellent assays; scores between 0 and 0.5 represent marginal assays; and scores less than 0 represent assays that are essentially impossible to use for screening purposes.

## Results

### Dose-Dependent Luciferase Expression

**Figure 1:**
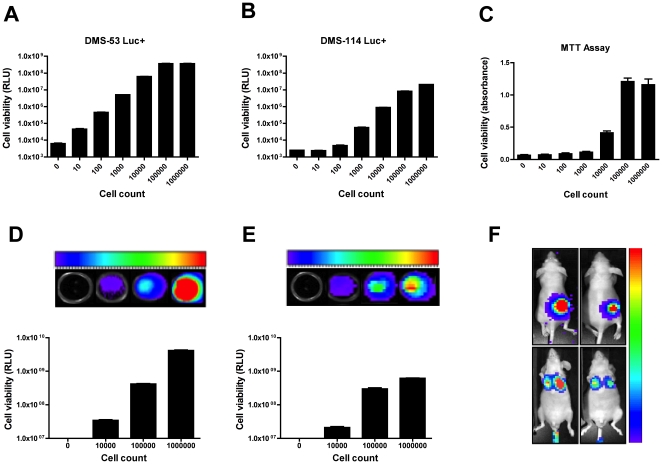
Establishment of luciferase-expressing SCLC cell lines for *in vitro* and *in vivo* assays. Serial dilutions of DMS-53 luc+ and DMS-114 luc+ cells were prepared, ranging from 0 to 1×10^6^ cells. Wells containing medium alone or 1×10^6^ DMS-53 and DMS-114 non-luciferase expressing cells were used as negative controls (0 cells). A traditional MTT assay was performed for comparison of sensitivity and dynamic range. Cell viability was measured using a luminometer (**A–B**), a spectrophotometer (**C**), or a Xenogen IVIS 100 imager (**D–F**). Colors represent clusters of CCD pixels while color scale represents luminescence intensity from lowest (violet) to highest (red). Instrument gain was set at min = 5×10^7^ photons/sec to max = 5×10^8^ photons/sec for DMS-53 luc+ *in vitro* (**D**) and at min = 25×10^6^ photons/sec to max = 25×10^7^ photons/sec for DMS-114 luc+ *in vitro* (**E**). Columns represent mean values and error bars represent standard error of means (n = 5 for luminometry, n = 8 for spectrophotometry, n = 4 for bioluminescence imaging). For *in vivo* imaging (**F**), mice were injected subcutaneously with DMS-53 luc+ (**upper left**) or DMS-114 luc+ (**upper right**) cells. For the lung colonization model, DMS-53 luc+ (**lower left**) or DMS-114 luc+ (**lower right**) cells were injected into the tail vein of mice. Instrument gain was set at min = 1×10^5^ photons/sec to max = 1×10^7^ photons/sec for the xenograft model and at min = 1×10^3^ photons/sec to max = 1×10^4^ photons/sec for the lung colonization model. **RLU** - relative luminescence units.

A lentiviral delivery approach was used to stably integrate a luciferase gene into the genome of two SCLC cell lines. Serial dilutions of these cells were then prepared to determine assay sensitivity. DMS-53 luc+ cells could be detected above background from as few as 10 cells using luminometry (Fig. 1A). Background readings were taken from wells containing medium alone or wells containing 1×10^6^ DMS-53 cells that do not express luciferase. The linear range of detection for DMS-53 luc + cells was between 1×10^1^ to 1×10^5^ cells. DMS-114 luc+ cells could be detected above background from as few as 100 cells (Fig. 1B). The linear range of detection for these cells was between 1×10^2^ to 1×10^5^ cells. For comparison, serial dilutions of DMS-53 luc+ cells were subjected to a traditional MTT assay (Fig. 1C). This approach required as many as 1×10^4^ cells to achieve absorbance values distinguishable from background. In addition, the linear range of detection for the MTT assay was only between 1×10^4^ and 1×10^5^ cells/well. Importantly, the MTT assay required at least 6 hours to run versus 45 minutes for the bioluminescence assay.

An additional advantage of using bioluminescent cell lines is their direct applicability to *in vivo* bioluminescence imaging. To confirm the utility of the luciferase-expressing cells for bioluminescence imaging, the Xenogen IVIS 100 imaging system was used, wherein the number of emitted photons is proportional to the number of bioluminescent cells. *In vitro*, the linear range of detection for DMS-53 luc+ was between 1×10^4^ and 1×10^6^ cells, yielding bioluminescence signals between 3×10^7^ to 4×10^9^ photons/sec (Fig. 1D). In comparison, the linear range of detection for DMS-114 luc+ was between 1×10^4^ and 1×10^5^ cells, yielding bioluminescence signals between 2×10^7^ and 3×10^8^ photons/sec (Fig. 1E). No luminescence signals could be detected in wells containing 1×10^6^ DMS-53 or DMS-114 cells that did not express luciferase. *In vivo*, DMS-53 luc+ and DMS-114 luc+ cells were used in a xenograft tumor model and a lung colonization model (Fig. 1F). For the xenograft model, 1×10^6^ DMS-53 luc+ and DMS-114 luc+ cells were detectable 15 minutes after injection of a luciferase substrate (upper left and right panels, respectively). Similarly, in the lung colonization model, 1×10^6^ DMS-53 luc+ and DMS-114 luc+ cells were detectable in the lung area after injection of a luciferase substrate (lower left and right panels respectively). Mice that were implanted with cells that do not express luciferase did not yield luminescence signals (data not shown).

### Response of Bioluminescent Cells to Pharmacological Agents and RNA Interference

**Figure 2:**
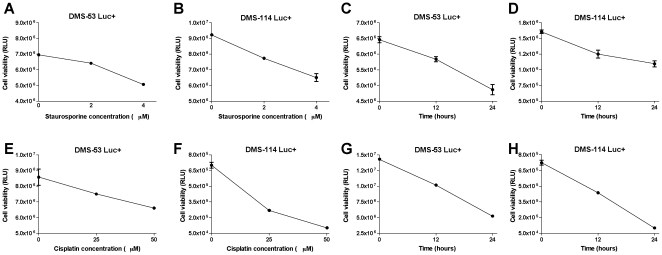
Bioluminescent SCLC cell lines respond to pharmacological agents in a dose- and time-dependent manner. DMS-53 luc+ and DMS-114 luc+ cells were treated with 0, 2, and 4 µM staurosporine, an apoptotic drug, for 24 hours (**A,B**) or with 4 µM staurosporine for 0, 12, and 24 hours (**C,D**). DMS-53 luc+ and DMS-114 luc+ cells were treated with 0, 25, and 50 µM cisplatin, a chemotherapeutic drug, for 24 hours (**E,F**) and 50 µM cisplatin for 0, 12, and 24 hours (**G,H**). Luciferase assays were then performed to measure cell viability. Data points represent mean values and error bars represent standard error of means (n = 5). **RLU** – relative luminescence units.

To test the hypothesis that luciferase expression reflects cell viability, we measured the responsiveness of the engineered SCLC cell lines to treatment with a known apoptosis-inducing agent, staurosporine. DMS-53 luc+ and DMS-114 luc+ cells were treated with staurosporine at varying doses (0, 2 and 4 µM) and time points (12 and 24 hours). As shown in Fig. 2A and 2B, luciferase activity of DMS-53 luc+ and DMS-114 luc+ cells decreased with increasing staurosporine concentration. Correspondingly, luciferase activity for both cell lines decreased with increased exposure time (Fig. 2C and 2D).

A similar strategy was employed to determine whether the engineered cells would also be responsive to a known chemotherapeutic agent, cisplatin. DMS-53 luc+ and DMS-114 luc+ cells were treated with cisplatin at varying doses (0, 25 and 50 µM) and time points (12 and 24 hours). An inverse relationship was observed between luciferase activity and cisplatin concentration (Fig. 2E and 2F). Similarly, luciferase activity decreased for both cell lines with increased exposure time (Fig. 2G and 2H).

**Figure 3:**
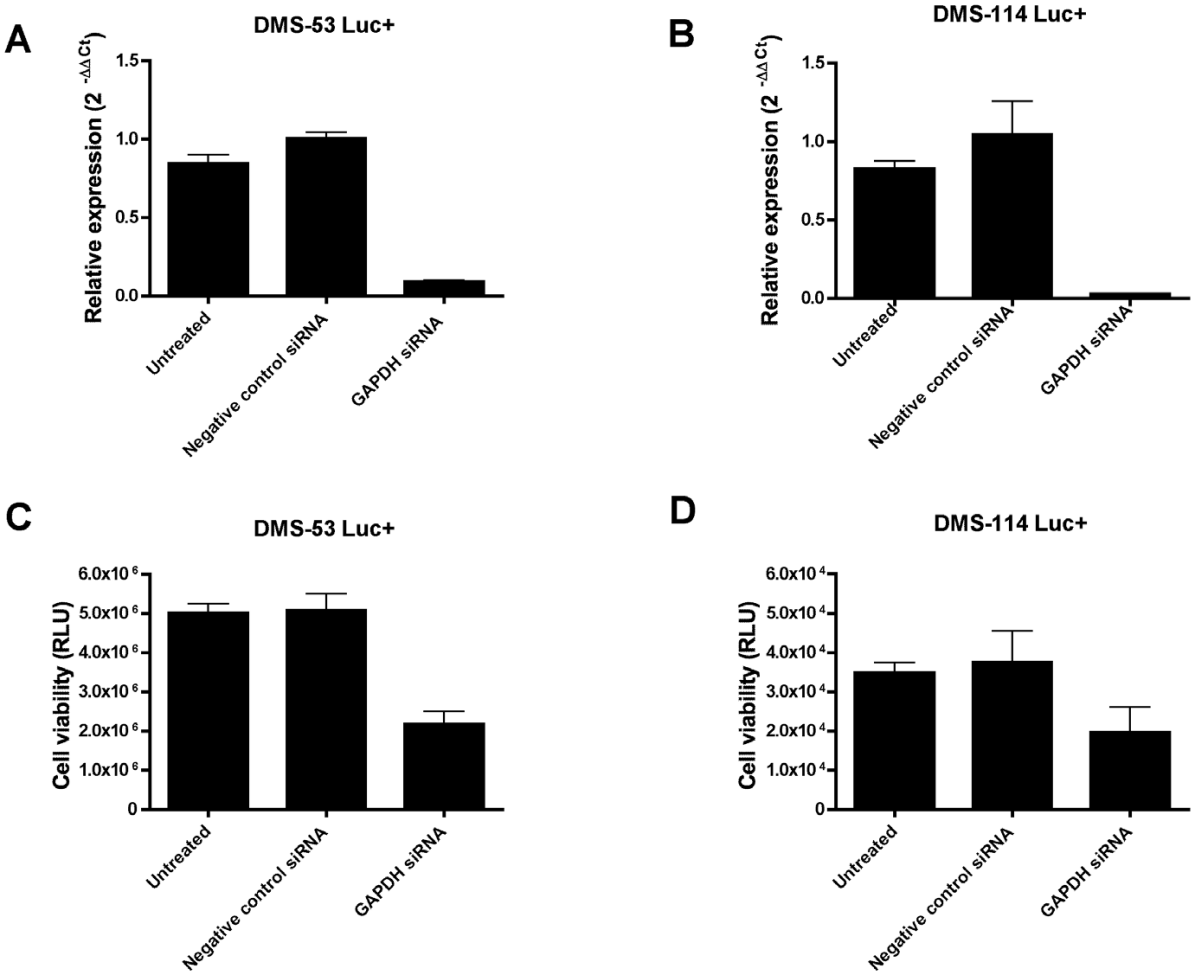
Bioluminescent SCLC cell lines respond to GAPDH depletion. DMS-53 luc+ and DMS-114 luc+ cells were treated with siRNAs against the housekeeping gene, GAPDH. GAPDH levels after knockdown were measured using quantitative RT-PCR. Approximately 91% and 97% knockdown was achieved for DMS-53 luc+ and DMS-114 luc+ cells, respectively (**A, B**). Negative controls included untreated cells (“Untreated”) and cells treated with a negative control siRNA provided by the manufacturer (“Negative Control siRNA”, Applied Biosystems). Cell viability upon GAPDH silencing was measured using luciferase assays (**C,D**). Points represent mean values and error bars represent standard error of means (n = 3 for quantitative RT-PCR, n = 4 for luciferase assays).

Finally, to test whether the viability of the engineered SCLC cell lines can be modulated by genetic manipulation, cells were treated with an siRNA against GAPDH, a known housekeeping gene. Knockdown levels of approximately 91% and 97% were achieved for DMS-53 luc+ and DMS-114 luc+, respectively (Fig. 3A and 3B). GAPDH knockdown resulted in decreased luciferase activity for both cell lines (Fig. 3C and 3D).

Taken together, these results indicate that the bioluminescence viability assay is a feasible assay for screening anti-SCLC therapies.

### High-Throughput Screening (HTS) of Compound Library

The bioluminescence viability assay protocol was modified for implementation in a high-throughput setting using the DMS-53 luc+ cell line. Assay quality was first verified using three different statistical parameters: S/B ratio, S/N ratio, and Z′-factors (see Materials and Methods). An S/B ratio of 3.1 and an S/N ratio of 18.6 were obtained. Both values lie within acceptable range (>2-fold). A Z′-factor value of 0.7 was also obtained, indicating that the assay was excellent for screening.

**Figure 4:**
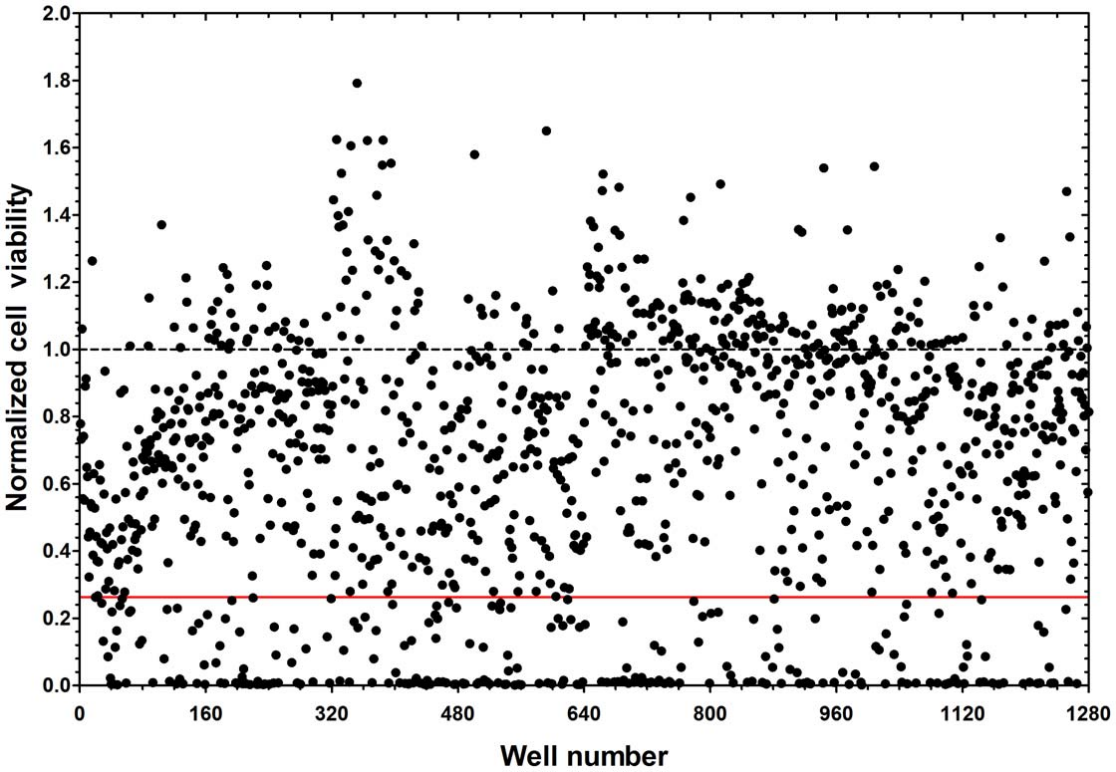
237 compounds inhibit SCLC cell viability. DMS-53 luc+ cells were treated with compounds from the LOPAC^1280^ library. Luciferase assays were then performed to measure the effect of the compounds on cell viability. Luciferase values were normalized to the mean luciferase values of the negative control, DMSO (dotted line). The solid red line indicates the mean value for the positive control, cisplatin. Compounds that resulted in inhibition greater than or equal to that of cisplatin were considered hits.

The assay was then used to evaluate a library of 1,280 compounds. In the primary screen, numerous compounds reduced cell viability (Figure 4). Compounds that reduced cell viability at an efficiency greater than or equal to cisplatin (∼77% reduction) were considered positive hits. A total of 237 hits were identified, comprising a diverse class of compounds (Table 1). The classes with the most number of hits (≥15) included compounds directed at phosphorylation, dopamine signaling and serotonin signaling.

**Table 1:** Classes of compounds that inhibit SCLC cell viability.

Class	Number of Hits
Adenosine	4
Adrenoreceptor	10
Angiogenesis	1
Antibiotic	2
Apoptosis	6
Benzodiazepine	1
Biochemistry	9
Ca^2+^ channel	10
Cannabinoid	2
Cell cycle	5
Cell stress	2
Cholinergic	7
Cytokines and growth factors	1
Cytoskeleton	4
DNA metabolism	3
Dopamine	27
GABA	5
Gene regulation	1
Glutamate	5
G-protein	4
Histamine	6
Hormone	7
Immune system	3
Intracellular calcium	4
Ion channels	2
Ion pump	5
K^+^ channel	5
Leukotriene	5
Lipid	4
Lipid signaling	2
Multi-drug resistance	2
Neurodegeneration	1
Neurotransmission	7
Nitric oxide	4
Opioid	5
P2 receptor	1
Phosphatase	1
Phosphorylation	39
Serotonin	15
Sphingolipid	1
Tachykinin	3
Transcription	3
Vanilloid	2
	Total = 237

Because phosphorylation is generally involved in a variety of physiological and pathological processes, we focused secondary screening on hits from the dopamine and serotonin classes of compounds. We retested these compounds first using DMS-53 luc+ cells. Of the 27 dopamine compounds, 24 were confirmed during secondary screening and of the 15 serotonin compounds, 12 were confirmed.

To ensure that reductions in viability caused by the various compounds were not specific for DMS-53 luc+ cells, the confirmed compounds were retested using DMS-114 luc+ cells. Of the 24 confirmed dopamine compounds, 22 caused reduction of viability in both DMS-53 luc+ and DMS-114 luc+ cells. Of the 12 confirmed serotonin compounds, all 12 reduced viability of DMS-53 luc+ and DMS-114 luc+ cells. Table 2 lists the compounds that were effective in reducing viability of both cell lines along with their specific pharmacological actions.

**Table 2:** Pharmacological agents that target neurotransmitter signaling in SCLC.

Class	Name	Action	Selectivity
Dopamine	BP 897	Agonist	D3
	Chlorprothixene hydrochloride	Antagonist	D2
	Cortexolone maleate*	Antagonist	D2
	(±)-Butaclamol hydrochloride	Antagonist	D2>D1
	R(+)-6-Bromo-APB hydrobromide	Agonist	D1
	BTCP hydrochloride	Blocker	Reuptake
	Chlorpromazine hydrochloride	Antagonist	-
	R(−)-N-Allylnorapomorphine hydrobromide	Agonist	-
	Dihydroergocristine methanesulfonate	Agonist	-
	R(−)-Propylnorapomorphine hydrochloride	Agonist	D2
	R(−)-2,10,11-Trihydroxyaporphine hybrobromide	Agonist	D2
	GBR-12909 dihydrochloride	Inhibitor	Reuptake
	R(−)-2,10,11-Trihydroxy-N-propylnoraporphine hydrobromide	Agonist	D2
	Fluspirilene	Antagonist	D2/D1
	cis-(Z)-Flupenthixol dihydrochloride	Antagonist	-
	Fluphenazine dihydrochloride	Antagonist	D1/D2
	GBR-12935 dihydrochloride	Inhibitor	Reuptake
	(±)-Octoclothepin maleate	Antagonist	D2
	Perphenazine	Antagonist	D2
	Pimozide	Antagonist	D2
	Prochlorperazine dimaleate	Antagonist	-
	Thiothixene hydrochloride	Antagonist	D1/D2
Serotonin	Amperozide hydrochloride	Ligand	-
	Paroxetine hydrochloride hemihydrate	Inhibitor	Reuptake
	CGS-12066A maleate	Agonist	5-HT1B
	S-(+)-Fluoxetine hydrochloride	Inhibitor	Reuptake
	Fluoxetine hydrochloride*	Inhibitor	Reuptake
	SB 228357	Antagonist	5-HT2B/2C
	Metergoline	Antagonist	5-HT2/5-HT1D
	GR 127935 hydrochloride hydrate	Antagonist	5-HT1B/1D
	Sertraline hydrochloride	Inhibitor	Reuptake
	Parthenolide	Inhibitor	-
	Ritanserin	Antagonist	5-HT2/5-HT1C
	SB 224289 hydrochloride	Antagonist	5-HT1B

*Representative drugs tested for tertiary verification.

- Unknown selectivity.

**Figure 5:**
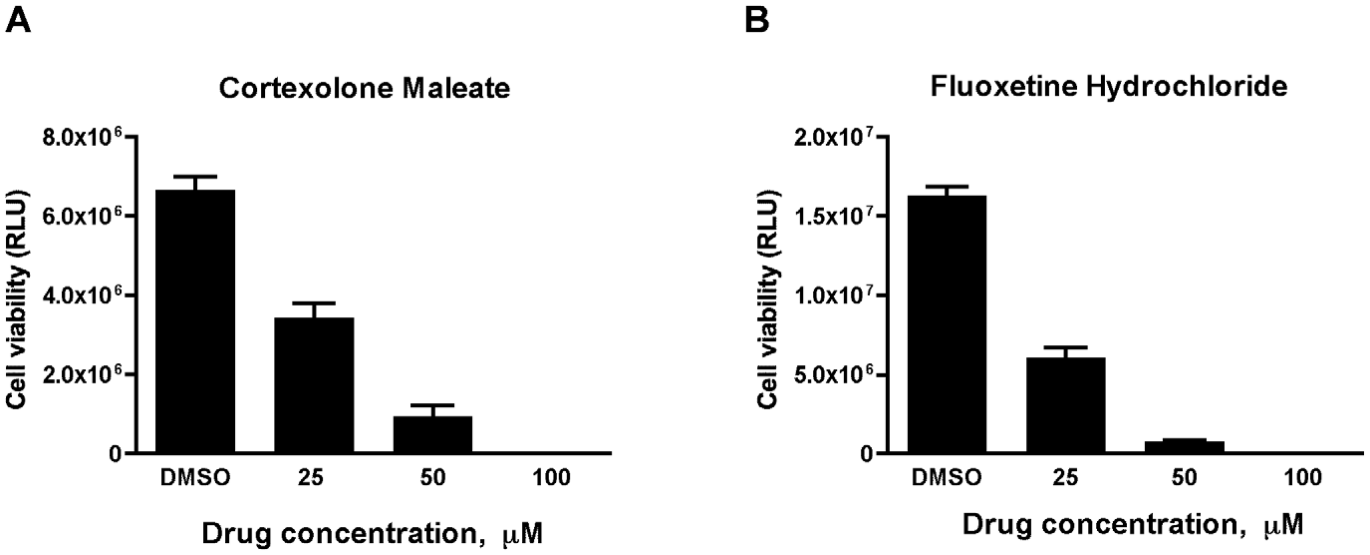
SCLC cells respond to cortexolone maleate and fluoxetine hydrochloride in a dose-dependent manner. DMS-53 luc+ cells were treated with 0, 25, 50, and 100 µM cortexolone maleate (**A**) or fluoxetine hydrochloride (**B**) for 24 hours. Luciferase assays were then performed to measure cell viability. Data points represent mean values and error bars represent standard error of means (n = 8). **RLU** – relative luminescence units.

Finally, for tertiary verification, we performed dose-response assays of two representative compounds, one from each class. As shown in Fig. 5A, treatment of DMS-53 luc+ cells with increasing doses of cortexolone maleate, a D_2_R dopamine receptor antagonist, resulted in corresponding decreases in cell viability. Similarly, increasing concentrations of fluoxetine hydrochloride, a selective serotonin reuptake inhibitor (SSRI), resulted in corresponding decreases in cell viability (Fig. 5B).

## Discussion

With the aim of uncovering novel therapeutic strategies against SCLC, we developed a bioluminescence-based cell viability assay for high-throughput screening of compound libraries. Phenotypic assays such as the one described here expedite primary screening of large numbers of chemicals, while limiting the use of animals in research. In this study, we used two cell lines, DMS-53 luc+ and DMS-114 luc+, that were originally derived from mediastinal biopsies of SCLC patients who had not received prior therapy, allowing delineation of specific effects of novel compounds [14]. We demonstrated broad dynamic range of detection for both cell lines. Increased sensitivity of the bioluminescence assay was also observed compared to a traditional MTT-based cell viability assay. Moreover, a direct relationship between luminescence signals and cell number was observed for both cell lines using two approaches, luminometry and bioluminescence imaging. The use of live animal bioluminescence imaging provides a more physiologically relevant context and allows for non-invasive, longitudinal monitoring of animals, again avoiding the use of large numbers of animals for research. These advantages notwithstanding, cell-based assays remain indispensable for large-scale screens.

Prior to performing such a screen, we assessed the responsiveness of the two engineered cell lines to standard pharmacological agents and RNA interference. Staurosporine, a member of the K252 family of compounds known to inhibit protein kinases [15], was used to show sensitivity of the engineered cells to an apoptosis-inducing drug. Cisplatin, a platinum-containing, broad activity anti-neoplastic and alkylating agent [16], was used to demonstrate the sensitivity of cells to a classic chemotherapeutic agent. Finally, RNA interference using siRNAs against GAPDH, a gene involved in vital metabolic functions [17], illustrated the utility of these cells for studies involving genetic treatments.

The assay was then implemented in a large-scale screen of the LOPAC^1280^ compound library. This library contains 1,280 pharmacologically active compounds. This annotated collection of small molecule modulators and FDA-approved drugs impacts most cellular processes and covers all major drug target classes. The LOPAC screen serves as an excellent starting point for validating high-throughput assays. Moreover, it potentially allows the identification of drugs that have available human dosage and toxicity information as well as the discovery of lead structures for drug development. Our primary screen identified several classes of drugs that reduced SCLC cell viability (Table 1). Of these, many have been implicated in fundamental processes associated with the etiology of cancer, such as angiogenesis, calcium signaling, cell cycle progression, and protein phosphorylation [18].

Interestingly, our screen identified several drug classes that impact neuroendocrine pathways known to be involved in SCLC pathogenesis. SCLC cells are characterized by neuroendocrine features such as the expression of ion channels, neuropeptides, and neurotransmitters and, as a consequence, are electrically excitable [19]. Here, we identified drugs that target adrenergic receptors, calcium channels, cholinergic receptors, dopamine signaling, GABA signaling, glutamate signaling, K^+^ channels, Na^+^ channels, opioid signaling and serotonin signaling [20].

We focused the follow-up screen on compounds that target dopamine and serotonin signaling as they yielded the highest number of hits. We did not pursue compounds in the protein phosphorylation class given the ubiquitous role protein phosphorylation plays in both normal and disease states [21], [22]. The secondary screening results essentially overlapped with those of the primary screen, indicating the reliability of the assay. Furthermore, the dose-dependent reduction in cell viability induced by the D_2_R antagonist, cortexolone maleate, and the SSRI, fluoxetine hydrocholoride, is consistent with the critical role of neurotransmitter signaling in the pathogenesis of SCLC [23].

Dopamine signaling has previously been implicated in SCLC [24]. In particular, the D_2_R agonist, bromocriptine, has been shown to have an anti-proliferative effect on SCLC cells *in vitro* and inhibits growth of SCLC tumor xenografts [25]. Unexpectedly, we observed that cortexolone maleate also has an anti-proliferative effect. These data suggest that the effect of bromocriptine in SCLC may be caused by D_2_R desensitization as opposed to agonism. In addition, serotonin has been shown to act as a mitogenic signal in SCLC, activating an autocrine growth loop in these cells [26], [27]. However, we found that fluoxetine hydrochloride, known to increase serotonin levels, inhibits SCLC growth. Another SSRI, imipramine, has previously been shown to have the same effect [28]. Taken together, these findings also posit ligand functional selectivity, a phenomenon wherein a drug acting through a single receptor can act as an agonist in some cases and as an antagonist in others [29].

In conclusion, we have described a bioluminescence-based assay for drug discovery in the field of SCLC therapeutics. Such an assay has not been previously applied to SCLC, a disease with very poor prognosis and limited treatment outcomes. The simplicity and speed of the workflow we developed not only allows for routine laboratory use but also lends itself to high-throughput applications and adaptability to automation. We have validated this assay against a library of pharmacologically active compounds. That positive hits included compounds targeting classic cancer signaling pathways suggests internal consistency. Compounds that target neurotransmission also emerged from the screen, reflecting the neuroendocrine nature of SCLC and underscoring the role of neurotransmitter signaling in this disease. In particular, perturbation of dopamine and serotonin signaling inhibits SCLC cell viability, suggesting the utility of these classes of drugs as therapeutic agents against SCLC.

## Supporting Information

Figure S1 DMSO tolerance of DMS-53 luc+ cells. Cells were treated either with 0.5% or 1% DMSO in complete medium for 24 hours. Cells in medium alone served as untreated controls. No significant difference in cell viability was observed after DMSO treatment. **RLU** – relative luminescence units.
(TIF)
